# Predicting the Dietary Metabolizable Energy and Crude Protein Levels of 1- to 70-Day-Old Hy-Line Brown Male Chicks Using Response Surface Methodology and Artificial Neural Network Models

**DOI:** 10.3390/ani16213395

**Published:** 2026-10-27

**Authors:** Heng Zhang, Xiangyu Zhang, Peipei Yan, Kun Zhang, Xuelan Liu, Chunyan Fu, Qingtao Gao, Yan Shang, Xia Li, Tianhong Shi

**Affiliations:** 1 Shandong Provincial Key Laboratory of Livestock and Poultry Breeding, Poultry Institute Shandong Academy of Agricultural Sciences, Jinan 250100, China; zhangheng2013@hotmail.com (H.Z.);; 2 Institute of Information Engineering, Chinese Academy of Sciences, Beijing 100085, China; 3 Shandong Animal Products Quality and Safety Center, Shandong Provincial Animal Husbandry and Veterinary Bureau, Jinan 250100, China

**Keywords:** Hy-Line Brown male chicks, RSM model, ANN model, multi-objective optimization

## Abstract

To determine dietary metabolizable energy (ME) and crude protein (CP) levels of Hy-Line Brown male chicks and compare response surface methodology (RSM) with artificial neural network (ANN) modeling, a total of 2160 birds were assigned to a 4 × 3 factorial design with three growth stages. Both RSM and ANN were fitted to predict body weight gain (BWG) and feed-to-gain ratio (F/G), with performance evaluated via LOOCV, leave-2-treatments-out validation, and bootstrap resampling. The 22–56-day stage yielded the best model performance overall. For F/G, both models achieved strong and comparable fit (in-sample R^2^ ≈ 0.86–0.88; LOOCV retention ≈ 97%), and both remained predictive under leave-2-treatments-out validation (RSM R^2^ = 0.72, ANN R^2^ = 0.45). For BWG, model performance was weaker, and ANN’s apparent in-sample advantage over RSM (R^2^ = 0.585 vs. 0.388 at 22–56 d) did not hold under leave-2-treatments-out validation (ANN R^2^ = −12.7 vs. RSM R^2^ = −0.24). Lastly, multi-objective optimization combining both models predicted stage-specific nutrient combinations. The results of this study provide empirical support and a methodological reference for establishing stage-specific nutritional standards for Hy-Line Brown male chicks.

## Simple Summary

Male chicks from layer breeds are often discarded at hatch, creating both economic waste and ethical concerns. Raising them for meat offers a way to reuse these birds, but the industry lacks clear feeding guidelines for them. We ran a 70-day feeding trial with 2160 Hy-Line Brown male chicks, testing four energy levels and three protein levels across three growth phases. Two modeling approaches—response surface methodology (RSM) and artificial neural networks (ANNs)—were compared for their ability to predict weight gain and feed efficiency. For F/G, both models performed well (R^2^ ≈ 0.86–0.88); for BWG, ANN’s in-sample advantage did not generalize under cross-validation (R^2^ = −12.7). RSM offered greater robustness under sparse data. Both models were ultimately used to predict optimal nutrient combinations, providing data support for establishing stage-specific nutritional standards for Hy-Line Brown male chicks.

## 1. Introduction

Hy-Line Brown male chicks are increasingly used as a niche broiler product in parts of China. They are valued for their disease hardiness, low input costs, and unique meat flavor [1,2]. Because no dedicated nutritional standards exist for these birds, producers default to generic broiler diets, which often produce uneven growth and poor feed conversion [3]. Developing a precision nutrition model is, therefore, a necessary step toward establishing feeding standards tailored to these birds.

Response surface methodology (RSM) is a common tool for optimizing biological systems. Its main strength is that it can tease apart complex relationships among multiple nutritional factors—both main effects and interactions—using relatively few experimental runs [4,5]. RSM is less effective, however, at capturing the highly nonlinear, dynamic interactions common in biological systems. This limitation has spurred interest in more flexible modeling approaches [6]. Artificial neural networks (ANNs) have attracted growing attention in poultry nutrition because they are naturally suited to complex nonlinear relationships. Researchers have applied them to model growth rate, feed intake, and environmental responses [7]. By learning connection weights and nonlinear activation functions, ANNs can map multiple dietary inputs (ME, CP, Ca, P) to production outputs such as body weight and feed intake with considerable accuracy [8,9]. A key bottleneck is that machine learning models require large, high-quality training datasets. In livestock production, breed-specific effects, fragmented farm settings, and the high cost of collecting labeled data all stand in the way of building models that generalize reliably [10].

Among the many nutritional factors in poultry diets, metabolizable energy (ME) and crude protein (CP) stand out as the most influential, and their interaction strongly shapes growth performance [11], jointly regulating growth rate and feed utilization efficiency. In practice, the energy-to-protein ratio must be matched to the breed, growth phase, and production goal. We, therefore, ran a three-phase feeding trial (1–21, 22–56, and 57–70 days of age) with graded levels of ME and CP. For each phase, we built both ANN and RSM models to identify the ME and CP combinations that maximize BWG and minimize F/G. Comparing the two approaches let us evaluate where each performs best. Beyond identifying the right model for each scenario, our results provide a foundation for setting nutrient levels of Hy-Line Brown male chicks across growth stages.

## 2. Materials and Methods

### 2.1. Experimental Design

All 2160 one-day-old Hy-Line Brown male chicks (Shandong Dezhou Braised Chicken Co., Ltd., Dezhou, China) were weighed individually at hatch. The chicks were first sorted by body weight and then randomly assigned within BW strata to the 12 treatment groups, with 6 replicates of 30 birds per group. The mean initial body weight for the 12 treatment groups ranged from 37.82 to 38.15 g, with a range of only 0.33 g between groups, and no significant differences among the groups (Table A3). The experiment was conducted over three stages: 1–21 days, 22–56 days, and 57–70 days of age. Detailed group assignments are presented in Table 1. A 4 × 3 two-factor experimental design was employed, with ME set at four levels and CP at three levels for each stage. For the 1–21-day stage, ME levels were 12.13, 11.92, 11.72, and 11.51 MJ/kg, and CP levels were 20%, 19%, and 18%. For the 22–56-day stage, ME levels were 12.13, 11.92, 11.72, and 11.51 MJ/kg, and CP levels were 19%, 18%, and 17%. For the 57–70-day stage, ME levels were 11.92, 11.72, 11.51, and 11.30 MJ/kg, and CP levels were 18%, 17%, and 16%. Experimental diets were formulated as corn–soybean meal-based diets with reference to the Feeding Standard of Chickens (NY/T 33-2004) [12]. The ingredient composition and nutrient levels of the basal diets for the 1–21-day, 22–56-day, and 57–70-day stages are presented in Table 2, Table A1, and Table A2, respectively. The animal trial was conducted at the experimental poultry house of the Poultry Research Institute, Shandong Academy of Agricultural Sciences. The experimental procedures were approved by the Institutional Animal Care and Use Committee of Shandong Academy of Agricultural Sciences (SAAS-2024-G21), approved on 25 April 2024.

### 2.2. Feeding Management

The experimental poultry house was a closed-type facility with automatic control of lighting, ventilation, and temperature, equipped with a manure belt for automatic manure removal. Birds were housed in H-type two-tier stacked cages, with 30 birds per cage as one replicate, and replicates of each treatment group were uniformly distributed throughout the house. Birds were provided with ad libitum access to feed and water. The ambient temperature was maintained at 35 °C from 1 to 7 days of age, and then reduced by 1 °C every 2 days until gradually reaching 25 °C. The relative humidity was controlled at 60–70%, with free access to water. The same birds and cages were used across all three stages, constituting a longitudinal design in which each cage progressed through the 1–21-, 22–56-, and 57–70-day diets sequentially.

### 2.3. Measurement Indices and Methods

#### Growth Performance

Residual feed and body weight of birds were accurately weighed at the beginning of the experiment (day 1) and at the end of each experimental stage (days 21, 56, and 70). The BWG and F/G for each stage were then calculated. Mortality was recorded daily throughout the trial, and birds that died were not replaced. No entire cage was lost; all 72 cages contributed data to all three feeding stages (Table A7). Feed intake was corrected to a bird-day basis, and F/G was computed as corrected feed intake divided by the body weight gain of the surviving birds.

BWG (g) = average body weight at the end of the stage − average body weight at the beginning of the stage

F/G = feed intake/body weight gain of the flock.

### 2.4. Modeling and Multi-Objective Optimization

RSM was fitted on all 72 cage-level observations using a second-order polynomial. The ANN was developed in MATLAB (R2022b; MathWorks, Natick, MA, USA). To prevent data leakage, 72 observations were split by treatment: eight combinations (48 observations) for training, two (12) for validation, and two (12) for testing, with all replicates of a given treatment kept together. Calculated formulation values were used for ME and CP inputs. Model performance was compared using MSE, MAE, and R^2^. Stability was assessed via LOOCV and 200-iteration bootstrap resampling.

#### 2.4.1. RSM Model

RSM construction and analysis were performed using Minitab (22.4.0; Minitab, LLC, State College, PA, USA) software. The second-order polynomial model was as follows:

Y = β_0_ + β_1_ME + β_2_CP + β_11_ME^2^ + β_22_CP^2^ + β_12_ME × CP + ε

where Y is the response variable (BWG or F/G); ME is dietary energy (MJ/kg); CP is dietary protein (%); β_0_ is the intercept; β_1_, β_2_ are linear coefficients; β_11_, β_22_ are quadratic coefficients; β_12_ is the interaction coefficient; and ε is random error.

A lack-of-fit test used within-treatment variation (6 replicates) to estimate pure error. Multiple-response optimization identified the maximum BWG and minimum F/G.

#### 2.4.2. ANN Modeling

**Figure 1:**
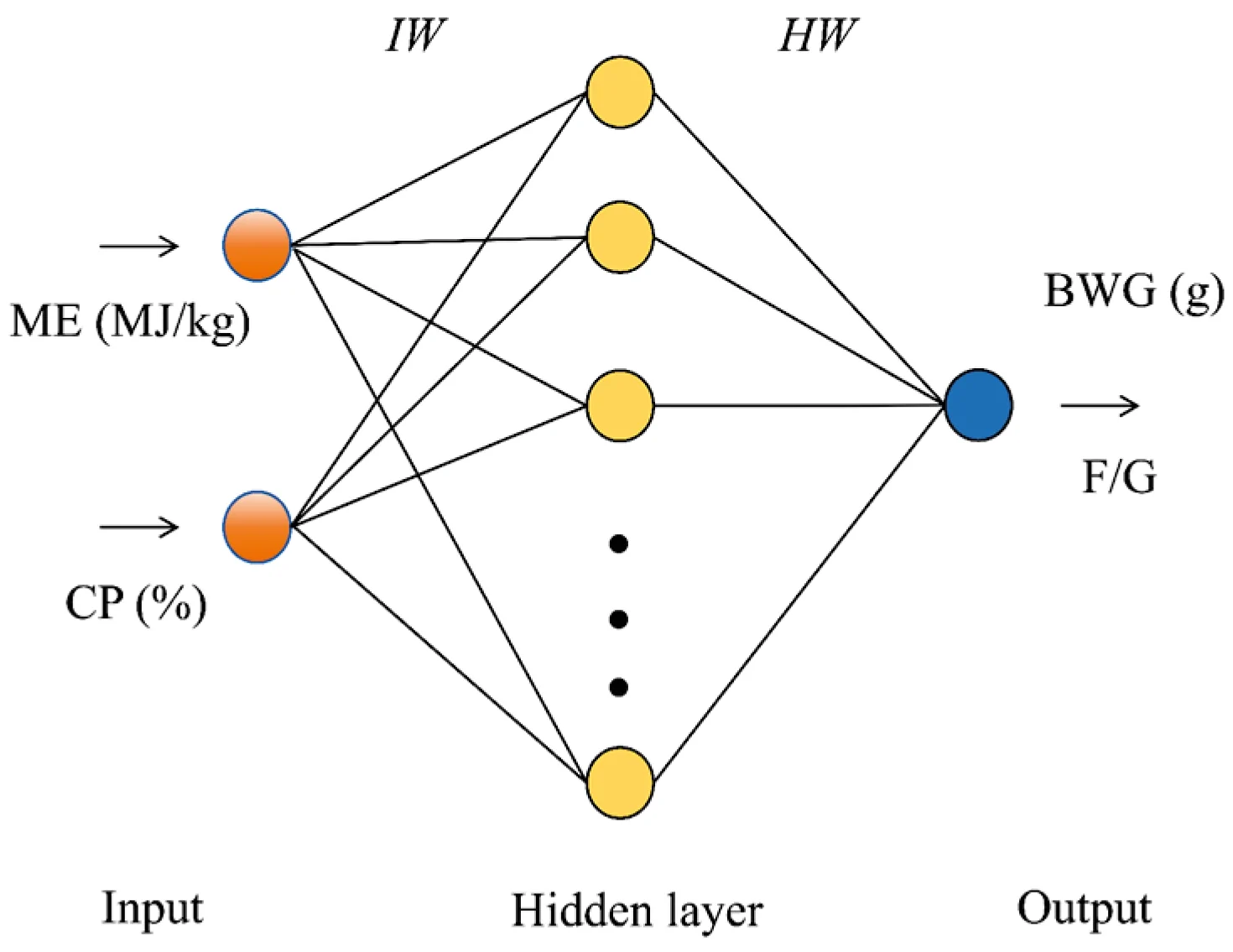
Schematic diagram of ANN architecture. Orange nodes (input layer): dietary ME and CP predictors. Yellow nodes (hidden layer): hidden neurons; the vertical ellipsis denotes additional hidden neurons not shown (node number is determined by hyperparameter tuning). Blue node (output layer): predicted BWG and F/G. Arrows indicate forward information flow. IW, input weights; HW, hidden weights.

We developed ANN models in MATLAB(R2022b) with the Neural Network Toolbox. Two separate networks (ANN-BWG and ANN-F/G) used a 2–5–1 architecture: 2 input neurons (ME, CP), 5 hidden neurons, and 1 output neuron (Figure 1). Both layers used log-sigmoid (logsig) transfer functions. To prevent data leakage, data were split by treatment: eight groups (1, 2, 3, 5, 6, 7, 9, 10) for training, two (4, 8) for validation, and two (11, 12) for testing. Each network had 21 trainable parameters (15 weights + 6 biases). Training used the Levenberg–Marquardt algorithm with early stopping based on validation MSE (6-epoch patience). The trained ANN served as the fitness function in an NSGA-II multi-objective optimization framework with BWG maximization and F/G minimization as objectives.

Pareto dominance was evaluated in the original (unnormalized) objective space—BWG in grams and F/G in g/g. Crowding distance was computed after normalizing both objectives to [0, 1] using each generation’s current population range (f’ i = (f i − f min)/(f max − f min)). This prevents BWG, which has a much larger numerical range, from dominating diversity maintenance. This per-generation renormalization is standard in NSGA-II. Final selection used the minimum-distance method on the input space (ME, CP), not the objective space. Both inputs were range-normalized to [0, 1] (ΔME = 0.62 MJ/kg; ΔCP = 2.0 percentage points), and normalized Euclidean distance to the model-predicted optimum was computed as follows:

Di = √[((Mei − MEopt)/ΔME)2 + ((Cpi − CPopt)/ΔCP)2]

NSGA-II parameters were set as follows: population size 200, maximum generations 200, crossover probability 0.8 (simulated binary crossover), mutation probability 1/*n*, and decision variables bounded by the experimental design ranges (Table A5). To assess sensitivity to the scaling method, the selection was repeated under z-score standardization and under no normalization; the selected treatment was identical across all three methods at every stage.

### 2.5. Bootstrap

To assess the stability of optima and Pareto front solutions, a nonparametric bootstrap was employed. For each stage, 72 cage-level observations were resampled with replacement to generate B = 200 bootstrap samples. For single-objective optimization, each sample was refitted by OLS using the same reduced-model terms as the final RSM, and the optimal ME–CP combination was identified on a 200 × 200 grid. For multi-objective optimization, two ANNs with the same architecture as the primary model (2–5–1, log-sigmoid, Levenberg–Marquardt, inputs normalized to [−1, 1]) were trained per sample using the full training protocol of the primary model (8-2-2 split by treatment, early stopping, lowest validation MSE selection). The original 8-2-2 treatment-to-set assignments were retained; when one or more treatment groups were absent from a bootstrap sample, the corresponding training, validation, or test subset was reduced accordingly. Bootstrap samples containing fewer than 10 distinct treatment combinations were discarded. The Pareto non-dominated set was identified, and a compromise solution was selected by minimum Euclidean distance to the ideal point after normalization. Selection frequencies and 95% bootstrap CIs were computed for optimal ME, CP, and Pareto solutions.

### 2.6. Statistical Analysis

(a) Stage-specific OLS models: Within each stage separately, OLS ANOVA (without prior-stage control) and OLS ANCOVA (with the preceding stage’s performance as a covariate) were fitted on 72 cage-level observations to provide cross-sectional inference.
(b) Wide-format mixed model (1|group): For stages 2 and 3, a wide-format mixed model was fitted with treatment group (*n* = 12) as a random intercept and the preceding stage’s performance as a fixed-effect covariate. Because each cage appears only once per stage in the wide-format data, no within-subject covariance structure was specified. Degrees of freedom were estimated using the Kenward–Roger method.
(c) Long-format mixed model (1|cage, primary model): A full longitudinal mixed model was fitted in long format using all three stages (216 observations from 72 cages, 3 observations per cage). Cage (*n* = 72) was specified as the repeated-measures subject with a random intercept and a compound symmetry (CS) covariance structure. The full model included fixed effects for ME, CP, stage, all two-way interactions (ME × CP, ME × stage, CP × stage), and the three-way interaction ME × CP × stage. The three-way ME × CP × stage interaction was non-significant for BWG (*p* = 0.080) but highly significant for F/G (*p* < 0.001). Following the principle of marginality, the term was retained for F/G only; final fixed effects differ by response, as detailed in Table A4.
  Neither 1|cage model includes a prior-stage performance covariate; cross-stage dependence is instead captured by the cage random intercept and CS covariance structure. These models serve as the primary inferential framework for diet effects.
(d) Interaction tests: Two interaction models were fitted to assess stage dependence of diet effects. Model A [long-format, no prior covariate, all 3 stages] tested ME × stage and CP × stage interactions (Table A4 Panel B). Model B [long-format, stages 2–3 only, with prior-stage covariate] tested the same interactions after explicit control for prior-stage performance (Table A4 Panel C).

The raw data, data preprocessing scripts, ANN training code, and optimization code are available in the Supplementary Materials (Zenodo: https://doi.org/10.5281/zenodo.22840312).

## 3. Results

### 3.1. Effects of Dietary Energy and Protein Levels on the Growth Performance of Hy-Line Brown Male Chicks

#### 3.1.1. Analysis of the Effects of Dietary Energy and Protein Levels on BWG of Hy-Line Brown Male Chicks

Table 3 summarizes the effects of dietary ME and CP on BWG and F/G (Table 3). During 1–21 days of age, the dietary ME level had no significant effect on BWG (*p* = 0.1125), whereas CP level had a highly significant effect (*p* < 0.0001), with the BWG of the 18% CP group being significantly lower than that of the 19% and 20% CP groups. During 22–56 days of age, ME level showed a highly significant effect on BWG (*p* < 0.0001), with the 11.92 MJ/kg group having the significantly highest BWG. CP level also showed a highly significant effect (*p* < 0.0001), with the BWG of the 17% CP group being significantly lower than that of the 18% and 19% CP groups. During 57–70 days of age, the effect of ME level on BWG did not reach a significant level (*p* = 0.0698), but CP level still had a highly significant effect on BWG (*p* < 0.0001), with the BWG of the 16% CP group being significantly lower than that of the 17% and 18% CP groups.

#### 3.1.2. Analysis of the Effects of Dietary Energy and Protein Levels on F/G of Hy-Line Brown Male Chicks

At 1–21 days, ME had no effect on F/G (*p* = 0.1225), while CP was significant (*p* = 0.0003). The 18% CP group had a higher F/G than the 19% and 20% CP groups.

#### 3.1.3. Analysis of the Interactive Effects of Dietary Energy and Protein Levels on the Growth Performance of Hy-Line Brown Male Chicks

At 22–56 days, the ME × CP interaction was highly significant for BWG (*p* = 0.0003): Group 6 outperformed all other groups, while Groups 11 and 12 had the lowest values. The ME × CP interaction for F/G was also highly significant at this stage (*p* = 0.0018); Group 12 had a higher F/G than all other groups. At 57–70 days, the interaction was significant for F/G (*p* = 0.0124). Groups 1 and 5 had lower F/G values than the remaining groups.

The longitudinal mixed model further revealed a significant ME × CP × stage three-way interaction for F/G (*p* < 0.001), with the ME × CP interaction strengthening progressively across stages (Table A4 Panel D). Because the three-way interaction was retained for F/G, the stage-specific ME × CP effects reported above should be interpreted as simple two-way effects at each stage rather than as overall (averaged) interaction terms.

### 3.2. Regression Analysis of the Effects of Dietary Energy and Protein Levels on the Growth Performance of Hy-Line Brown Male Chicks

We fitted quadratic polynomial response surface models with ME and CP as independent variables for BWG and F/G at each growth stage. All six regression models reached a highly significant level (*p* < 0.001), and the lack-of-fit test *p*-values (*p*_LOF) ranged from 0.234 to 0.942, all greater than 0.05, indicating that the models had good fit with no significant lack of fit and could effectively describe the effects of ME and CP levels on growth performance (Table 4).

The BWG_1–21_ model showed significant linear and quadratic effects of both ME and CP (ME^2^ and CP^2^ both negative), yielding a concave surface. The ME × CP interaction was not significant in either model (*p* = 0.763 for BWG, *p* = 0.404 for F/G), consistent with ME and CP acting largely independently at this stage. For F/G, only the CP^2^ term reached significance (*p* = 0.008).

For BWG_22–56_, CP linear (*p* = 0.020) and CP^2^ (*p* = 0.001, negative) were significant, whereas ME linear (*p* = 0.311), ME^2^ (*p* = 0.289), and ME × CP (*p* = 0.708) were not, indicating weak curvature in the ME dimension and a near-linear BWG response to energy; the negative CP^2^ placed the theoretical CP optimum at 18.28%. For F/G, all terms were significant (*p* ≤ 0.010), including a positive ME × CP interaction (0.074); however, the high multicollinearity of uncentered polynomial terms cautions against interpreting coefficient signs as biological synergism.

For BWG_57–70_, CP linear (*p* < 0.001), CP^2^ (*p* = 0.002, negative), and ME × CP (*p* = 0.046, −12.95) were significant, while ME linear (*p* = 0.627) and ME^2^ (*p* = 0.891) were not. The negative ME × CP interaction was the largest across stages, suggesting the ME–CP interplay strengthens with age. For F/G, CP linear (*p* < 0.001, negative), CP^2^ (*p* < 0.001, positive), and ME × CP (*p* < 0.001, positive) were significant, whereas ME linear (*p* = 0.397) and ME^2^ (*p* = 0.888) were not; all terms were retained to support the full second-order structure used by the response optimizer.

Full quadratic models (Constant + ME + CP + ME^2^ + CP^2^ + ME × CP) were fitted for all six stage–response combinations. All polynomial terms were retained regardless of statistical significance to maintain model hierarchy and ensure consistent comparison across stages. Complete coefficient tables, including estimates, standard errors, t-values, *p*-values, 95% confidence intervals, and variance inflation factors, are provided in Table A6. All six models satisfied assumptions of normality (Shapiro–Wilk test, all *p* > 0.05) and had no influential outliers (Cook’s distance < 4/*n* for all observations).

### 3.3. ANN Modeling of the Effects of Dietary Energy and Protein Levels on the Growth Performance of Hy-Line Brown Male Chicks

We trained ANN models with ME and CP as inputs to predict BWG and F/G at each growth stage. Fit was assessed by the correlation coefficient (R) for the training, validation, test, and combined datasets (Table 5). At 1–21 days, the BWG model had an overall R of 0.784 (training R = 0.817, validation R = 0.702, test R = 0.734), pointing to moderate association between predicted and observed BWG. The F/G model showed a weaker association (overall R = 0.651, validation R = 0.555), suggesting feed efficiency is harder to predict at this early age. At 22–56 days, the models performed best. The BWG model gave an overall R of 0.804, an improvement over the 1–21-day phase. The F/G model had the strongest fit of any stage: overall R = 0.941, training R = 0.964, validation R = 0.913, test R = 0.936. The close agreement among training, validation, and test R values suggests that the association between predicted and observed F/G was stable across data subsets. At 57–70 days, the F/G model remained strong (overall R = 0.925). The BWG model, however, had the weakest fit of any stage (overall R = 0.687).

Note on ANN evaluation levels. The ANN model was evaluated at three distinct levels across this study. (1) Cage-level single split (Table 5): the ANN was trained on 48 cages, validated on 12, and tested on 12, with Pearson correlation coefficients (R) reported for individual cage observations. (2) Treatment-mean level (Table 6): predictions from the same cage-level ANN were averaged to the 12 treatment means and compared with RSM predictions at the design-point level via R^2^, MSE, and MAE. (3) Cage-level resampling (Table 7): in-sample R^2^ and cross-validation R^2^ (LOOCV and leave-2-treatments-out) were computed across all 72 cage observations to assess generalizability at the individual replicate level. Treatment-mean-level R^2^ is typically higher than cage-level R^2^ because within-treatment replicate variation is averaged out; Table 5 reports correlation coefficients (R) rather than coefficients of determination (R^2^), so the three tables each answer a different question about model performance.

### 3.4. Comparative Evaluation of Model Performance Between RSM and ANN

To enable a direct head-to-head comparison at the same aggregation level, both model predictions were evaluated at the treatment-mean level (*n* = 12 design points). Table 6 compares ANN and RSM predictions using R^2^, MSE, and MAE. At 1–21 days, RSM and ANN were close for BWG (R^2^ = 0.624 vs. 0.615), with RSM showing lower MSE (0.258 vs. 0.897) and MAE (0.448 vs. 0.703). For F/G, ANN had a slightly higher R^2^ (0.420 vs. 0.403) and comparable MSE (1.34 × 10^−4^ vs. 1.36 × 10^−4^) and MAE (0.0097 vs. 0.00985). At 22–56 days, ANN achieved higher R^2^ for BWG: R^2^ = 0.645 vs. 0.480, with much lower MSE (12.107 vs. 30.25) and MAE (2.240 vs. 4.286). For F/G, ANN showed a slightly higher R^2^ (0.885 vs. 0.862) and lower MSE (2.75 × 10^−4^ vs. 3.3 × 10^−4^) and MAE (0.0137 vs. 0.0150) than RSM. At 57–70 days, ANN showed modestly higher R^2^ for BWG (R^2^ = 0.472 vs. 0.446), with lower MSE (4.748 vs. 13.527) and MAE (0.959 vs. 3.085). For F/G, the two models were nearly identical in R^2^ (0.848 vs. 0.846). ANN had slightly higher MSE (5.12 × 10^−4^ vs. 5.05 × 10^−4^) and comparable MAE (0.0194 vs. 0.0193). The ANN model’s highest in-sample R^2^ was observed for BWG at 22–56 days and was competitive with RSM on F/G at 1–21 days. For BWG at 1–21 days and F/G at 57–70 days, the two methods had complementary strengths. Neither model was uniformly better across all stage–response combinations.

Note that RSM and ANN R^2^ values in Table 6 are computed at the treatment-mean level (*n* = 12); an equivalent cage-level comparison (*n* = 72) is provided in Table 7.

### 3.5. Cross-Validation and Resampling

The LOOCV results (Table 7) showed that the F/G models retained most of their in-sample R^2^: 97% for RSM and ANN at 22–56 d (0.862 → 0.838 and 0.882 → 0.851) and 90–94% at 57–70 d (0.764 → 0.717 and 0.781 → 0.700). At 1–21 d, retention was lower for F/G (53–61%), reflecting the already modest in-sample R^2^ (0.273–0.286) at that stage. For BWG, LOOCV retention ranged from 47% (ANN, 57–70 d) to 78% (RSM, 1–21 d).

The leave-2-treatments-out analysis produced the widest gap between the two model classes. For BWG, the ANN R^2^ dropped to −1.402 (1–21 d), −12.682 (22–56 d), and −7.172 (57–70 d), while the RSM values were 0.376, −0.235, and −0.237. For F/G, both models remained positive at 22–56 d and 57–70 d, with RSM at 0.721 and 0.600 and ANN at 0.447 and 0.649.

The bootstrap analysis results indicate that ANN-based Pareto compromise solutions (Table A8) were highly stable for 22–56 d (selection frequency 98.0%; compromise Group 6, ME = 11.92 MJ/kg, CP = 18%) but less stable for 1–21 d (24.5%; compromise Group 2, ME = 11.92 MJ/kg, CP = 20%) and 57–70 d (57.5%; compromise Group 1, ME = 11.92 MJ/kg, CP = 18%). The lower stability for 1–21 d reflected divergent single-objective optima (BWG optimum at Group 6, CP = 19%; F/G optimum at Group 5, ME = 12.13 MJ/kg), creating tension in the trade-off. The 95% bootstrap CIs for compromise ME were [11.72, 12.13], [11.92, 11.92], and [11.50, 11.92] MJ/kg for 1–21 d, 22–56 d, and 57–70 d, respectively; corresponding CP CIs were [19.00, 20.00], [18.00, 18.00], and [17.00, 18.00]%.

Prediction uncertainty was propagated to the Pareto front via bootstrap resampling. In conjunction with cross-validation results, the F/G Pareto front was most stable for the 22–56 d and 57–70 d periods, consistent with its LOOCV retention rates of 94–97% and positive out-of-sample R^2^ values (ANN: 0.447 and 0.649, respectively). In contrast, the BWG front exhibited lower stability. Across all three stages, the selected dietary regimens were predominantly governed by the F/G criterion, as this criterion proved more reliable than the alternative in the present dataset.

### 3.6. Predicting the ME and CP Levels of Hy-Line Brown Male Chicks Using RSM and ANN

**Figure 2:**
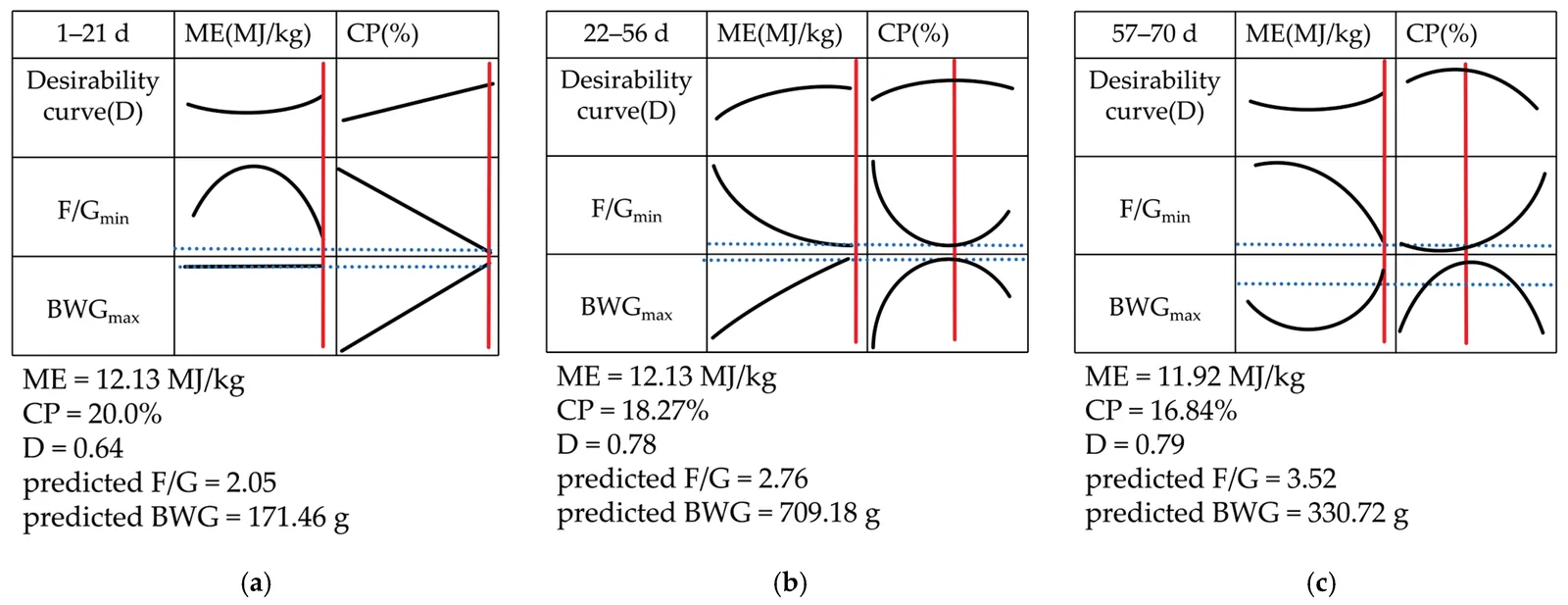
Multi-response optimization plots from RSM models, showing the model-optimized ME and CP levels that simultaneously maximize BWG and minimize F/G for Hy-Line Brown male chicks at three growth stages. Each panel is a 3 × 2 grid generated by the Minitab (22.4.0) Response Optimizer. The left column plots responses against ME (MJ/kg); the right column plots responses against CP (%). The top row shows the composite desirability function (D, 0–1 scale); the middle row shows the predicted F/G (minimized, y-axis = feed-to-gain ratio); the bottom row shows the predicted BWG (maximized, y-axis = g/bird). In each subplot, the grey curve is the predicted response profile across the tested factor range; the red vertical line marks the optimal factor setting; and the blue dashed horizontal line marks the predicted response value at the optimum. The model-optimized values are labelled in the column header (top) and row labels (left margin). (**a**) 1–21 d: ME = 12.13 MJ/kg, CP = 20.0%, D = 0.64, predicted F/G = 2.05, predicted BWG = 171.46 g. (**b**) 22–56 d: ME = 12.13 MJ/kg, CP = 18.27%, D = 0.78, predicted F/G = 2.76, predicted BWG = 709.18 g. (**c**) 57–70 d: ME = 11.92 MJ/kg, CP = 16.84%, D = 0.79, predicted F/G = 3.52, predicted BWG = 330.72 g.

**Figure 3:**
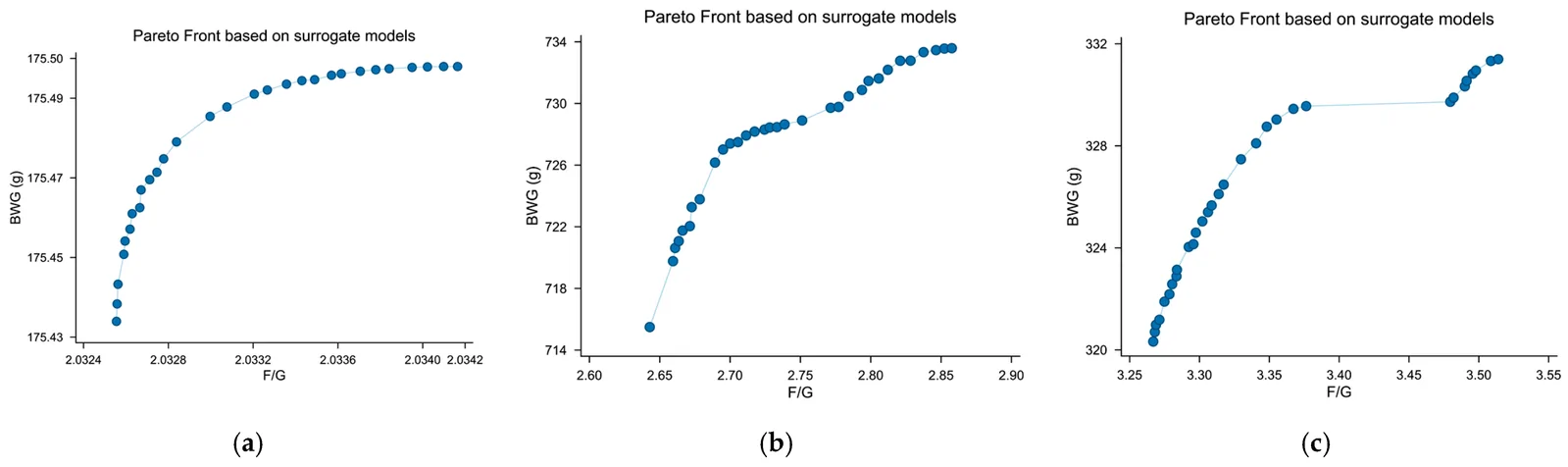
Pareto fronts derived from the ANN models, showing the trade-off between F/G (x-axis) and BWG (g/bird, y-axis) for Hy-Line Brown male chicks at three growth stages. Each blue circle represents a non-dominated solution, where improving one objective requires sacrificing the other; the connecting line traces the Pareto front. (**a**) 1–21 d: ME = 11.89 MJ/kg, CP = 19.50%, BWG = 175.47 g, F/G = 2.03. (**b**) 22–56 d: ME = 11.92 MJ/kg, CP = 18.0%, BWG = 724.14 g, F/G = 2.78. (**c**) 57–70 d: ME = 11.91 MJ/kg, CP = 16.83%, BWG = 328.72 g, F/G = 3.34.

Multi-objective optimization of BWG and F/G via the response optimizer gave the following ME and CP combinations (Figure 2): For 1–21 days, the model-optimized combination was ME = 12.13 MJ/kg and CP = 20.0%, giving predicted BWG = 171.46 g and F/G = 2.05 (composite desirability D = 0.64). For 22–56 days, the optimum was ME = 12.13 MJ/kg and CP = 18.27%, with predicted BWG = 709.18 g and F/G = 2.76 (D = 0.78). For 57–70 days, the optimum was ME = 11.92 MJ/kg and CP = 16.84%, with BWG = 330.72 g and F/G = 3.52 (D = 0.79; Figure 2). At all three stages, the RSM-optimized ME coincided with the highest tested level, meaning these are boundary optima rather than interior optima; the true ME optimum may lie above the tested range. Figure 3 shows the Pareto fronts derived from the ANN surrogate models. These fronts illustrate the trade-off between maximizing BWG and minimizing F/G at each stage. Projecting the ANN-optimized combinations onto these fronts provides a visual check of how well they balance the two objectives. At 1–21 days, the Pareto front spans F/G = 2.0324–2.0342 and BWG = 175.43–175.50 g, with a concave asymptotic shape. The ANN-optimized combination (ME = 11.89 MJ/kg, CP = 19.50%) gives BWG = 175.47 g and F/G = 2.03. This point sits in the upper-left region of the front, confirming that both objectives are nearly optimized at once. The RSM solution (ME = 12.13 MJ/kg, CP = 20.0%) gave BWG = 171.45 g and F/G = 2.04, lower BWG at a similar F/G, suggesting the ANN combination uses energy more efficiently. At 22–56 days, the Pareto front spans F/G = 2.63–2.87 and BWG = 715–733 g. The curve has a three-stage shape. The ANN-optimized combination (ME = 11.92 MJ/kg, CP = 18.0%) gives BWG = 724.14 g and F/G = 2.78. This point sits in the transition from the plateau to the secondary rise of the front, with a slightly higher F/G. The response optimizer (ME = 12.13 MJ/kg, CP = 18.27%) gives BWG = 709.18 g and F/G = 2.76—slightly better F/G but notably lower BWG. Though the ANN point had a slightly higher F/G, the gain in BWG (+14.96 g) makes it the stronger choice in Pareto terms. At 57–70 days, the Pareto front spans F/G = 3.26–3.53 and BWG = 320–331 g. The curve is clearly piecewise; a visible gap probably reflects sparse data in that region of the surrogate model. The ANN-optimized combination (ME = 11.91 MJ/kg, CP = 16.83%) gives BWG = 328.72 g and F/G = 3.34. This point lies in the middle-to-rear portion of the left segment, with BWG approaching the upper limit of that segment (330 g) and a moderate F/G. The response optimizer (ME = 11.92 MJ/kg, CP = 16.84%) gives BWG = 330.71 g and F/G = 3.51— BWG is less than 2 g higher, but F/G is worse by 0.17. The ANN point, lying on the left segment and avoiding the gap, achieves near-maximum BWG at a modest cost in F/G.

Across all three stages, the ANN-optimized combinations fell on or near the non-dominated region of the Pareto front. At 1–21 days, the ANN point was closest to the front, in a favorable position on the front. At 22–56 days, the ANN point sat in the middle segment, striking a good balance between the two objectives. At 57–70 days, the ANN point avoided the data gap and fell on the reliable left segment. Notably, all three ANN-predicted ME values fell inside the tested range rather than at its boundary, in contrast to the RSM boundary optima.

## 4. Discussion

### 4.1. Study on the Nutritional Levels of Hy-Line Brown Male Chicks

We used both ANN and RSM to estimate the model-optimized ME and CP levels for Hy-Line Brown male chicks across three growth stages. These findings can guide diet formulation and improve the production efficiency of these birds. Research on the nutrient levels of Hy-Line Brown male chicks is still scarce. In existing studies, Huang et al. [17] designed diets with high energy and high protein (ME of 12.55 and 13.22 MJ/kg, CP of 21% and 18%, respectively) and low energy and low protein (ME of 12.17 and 12.36 MJ/kg, CP of 20% and 17.5%, respectively) to study the growth and development patterns of Hy-Line Brown male chicks under different nutritional levels. They fitted three nonlinear models—Logistic, Gompertz, and Von Bertalanffy—to body weight data from 1 to 12 weeks, providing a preliminary look at growth patterns under different diets. Yao et al. [18] further investigated the effects of different energy and protein feed formulations on growth performance, slaughter performance and other indicators, providing a reference for feed formula screening. To build a more systematic feeding program, we extended the rearing period to 70 days across three consecutive phases. We split the trial into three feeding phases—starter (0–3 wk), grower (3–8 wk), and finisher (9–10 wk)—drawing on growth curve data, published international work, and the raw-material specifications of the Dezhou braised chicken trade. Huang et al. [17], fitting Gompertz curves to Hy-Line Brown males, put the growth inflection point at 7.64 to 8.66 weeks (53.5–60.6 days); it is because this peak sits late in week 8 that we let the grower phase run a full five weeks, so that the entire acceleration period would sit within one dietary stage. Popova et al. [19], working with Lohmann Brown Classic males on a standard broiler diet, recorded around 1.1 kg at 9 weeks, which lines up with a 9- to 10-week market window for layer-type males. On the production side, Dezhou braised chicken calls for birds in the 1.0–1.5 kg range, with tender flesh and moderate fat; under northern Chinese feeding conditions, that specification is met at roughly 70 days, and the 14-day finisher that closes the trial gives time to step protein down and let energy drive deposition and meat quality.

### 4.2. Carry-Over Effects and the Longitudinal Feeding Design

The same birds and cages were used across all three feeding stages, a design that introduces statistical dependence between stages. To assess whether this carry-over inflates the apparent diet effects, we re-analyzed each later stage with the preceding stage’s performance as a covariate (Table A4). At 22–56 d, after controlling for the 1–21 d F/G as a covariate, F/G remained significantly associated with both current dietary ME (*p* = 0.011) and CP (*p* = 0.018). The prior-stage F/G covariate was itself significant (*p* = 0.024), confirming that carry-over exists but does not erase the current-diet signal. Under the wide-format group-random-intercept model (1|group), ME (*p* = 0.283) and CP (*p* = 0.326) were no longer significant; however, this model is overly conservative because each of the 12 treatment groups corresponds to a unique ME × CP combination, creating complete structural nesting of diet effects within between-group variability. Under the long-format cage-random-intercept model (1|cage), which retained the significant ME × CP × stage three-way interaction, the simple main effects of ME and CP for F/G at 22–56 d were highly significant (*p* < 0.001 for both), reflecting the stage-dependent nature of the diet effects. At 57–70 d, both effects remained highly significant (*p* < 0.001 for both). The discrepancy between the two model specifications underscores the importance of using cage as the repeated-measures subject in longitudinal feeding trials. At 57–70 d, the picture was similar for F/G—ME and CP remained significant under both the OLS ANCOVA (*p* = 0.002 and 0.005) and the 1|group model (*p* = 0.007 and 0.015), and the prior F/G was significant (*p* = 0.019). For this stage–response combination, all four analytical approaches (OLS ANOVA, OLS ANCOVA, 1|group, and 1|cage) converged on the same conclusion. Notably, the F/G mixed model retained a significant ME × CP × stage three-way interaction (*p* < 0.001; Table A4 Panel D), with simple ME × CP interactions strengthening from non-significant at 1–21 d (*p* = 0.457) to marginal at 22–56 d (*p* = 0.074) and highly significant at 57–70 d (*p* < 0.001), consistent with a developmental shift in energy–protein coordination.

For BWG at 57–70 d, controlling for the 22–56 d BWG appeared to sharpen the diet effects: ME and CP moved from marginally non-significant (*p* ≈ 0.053 and 0.056) to nominally significant (*p* = 0.031 and 0.029), while the prior-stage BWG was highly significant (*p* = 0.0003). Under the 1|group model, these effects were attenuated below significance (ME: *p* = 0.094; CP: *p* = 0.088). In contrast, under the 1|cage model, ME was significant (*p* = 0.009) and CP was highly significant (*p* < 0.001). The attenuation in the 1|group model reflects the complete structural nesting of diet within treatment group (each of the 12 groups maps to a unique ME × CP combination), which absorbs diet variance into group variance and reduces the effective degrees of freedom for testing diet effects, rather than a true loss of signal, as the 1|cage model confirms that dietary ME and CP exert significant effects on BWG at this stage after accounting for within-cage correlation across stages via a cage random intercept and compound symmetry covariance structure. It should be noted that the 1|cage model does not include an explicit prior-stage performance covariate; the OLS ANCOVA (which does include such a covariate) also detected nominally significant effects of ME (*p* = 0.031) and CP (*p* = 0.029) at this stage, providing convergent evidence. For BWG at 22–56 d, neither ME nor CP reached significance under the OLS ANCOVA or the 1|group model, consistent with the compensatory growth patterns observed in male broilers during the grower phase [20,21]. However, under the 1|cage model, ME was marginally significant (*p* = 0.03) and CP was also marginally significant (*p* = 0.05). This divergence indicates that the grower-phase BWG response to dietary ME and CP is detectable when the longitudinal structure is modelled with cage as the repeated-measures subject (which borrows information across 216 observations and accounts for within-cage correlation) but is obscured by the 1|group specification in which diet is structurally nested within group. Notably, only the 1|cage model detected significance for this stage–response combination; the OLS ANOVA (*p* = 0.874/0.783), OLS ANCOVA (*p* = 0.845/0.944), and 1|group model (*p* = 0.936/0.894) all yielded non-significant results. The 1|cage model’s greater statistical power stems from pooling all three stages in a single model, but this design also means that stage-specific conclusions for 22–56 d BWG rely on a model whose primary evidence comes from cross-stage correlation borrowing rather than within-stage signal alone. The biological interpretation is nuanced: compensatory growth may dampen the magnitude of diet effects on BWG during this phase (as reflected in the smaller effect sizes and non-significance under OLS) but does not eliminate them entirely (as shown by the 1|cage model).

Across all four stage–response combinations, the 1|cage and 1|group models yielded qualitatively different conclusions in three instances (22–56 d BWG, 22–56 d F/G, and 57–70 d BWG), with the 1|cage model detecting diet effects (marginally significant for 22–56 d BWG; significant for the other two) that the 1|group model missed; only 57–70 d F/G showed convergence between the two specifications. The model specifications are detailed in Section 2.6. In the OLS ANCOVA (which includes an explicit prior-stage performance covariate), the prior-stage carry-over was statistically significant for F/G (*p* = 0.024 at 22–56 d; *p* = 0.019 at 57–70 d) and showed a mixed pattern for BWG (*p* = 0.087 at 22–56 d; *p* = 0.0003 at 57–70 d), suggesting that feed efficiency in later stages depends on earlier growth trajectories, whereas body weight gain shows a more complex carry-over pattern—non-significant at 22–56 d but highly significant at 57–70 d. The long-format 1|cage model does not include an explicit prior-stage covariate; instead, cross-stage dependence is captured through the cage random intercept. This differential carry-over pattern has practical implications for phase-feeding strategies: adjusting ME and CP for F/G must consider prior-stage performance, while BWG responses are largely independent of previous growth. Notably, the ME × stage and CP × stage interactions for BWG were significant in Model A (Panel B, *p* = 0.001 and 0.009) but became non-significant in Model B (Panel C, *p* = 0.247 and 0.402) after controlling for prior-stage performance, suggesting that part of the apparent stage dependence may be attributable to carry-over rather than genuine stage-specific diet responses.

In addition, the cumulative nature of nutrient intake is not a nuisance to be eliminated but part of the biological signal. Prior-stage nutrition shapes organ development, gut maturation, and metabolic capacity in ways that carry into subsequent stages [22,23], so a design that severs that continuity would produce dietary-level estimates applicable only to birds with no feeding history—a situation that never occurs in commercial production. The model-estimated dietary levels reported in this study should, therefore, be read as the model-optimized ME and CP levels for birds on a continuous nutritional trajectory from hatching to market, which is the trajectory that actually exists in practice.

### 4.3. Model Construction

RSM is a well-established tool for optimizing dietary nutrient levels in poultry. It uses a polynomial model that captures main effects and interactions to quantify how nutrient levels relate to growth performance. Ghazaghi et al. [24] used the RSM model to determine the dietary ME and CP levels of quail, and the regression equation for body weight gain (BWG) with ME and CP levels was as follows: BWG = 407.3 − 0.24ME − 2.3CP + 0.00003ME^2^ + 0.001ME × CP − 0.002CP^2^. In another study on broilers, researchers [25] used the RSM model to observe the effects of dietary digestible calcium levels on body weight gain, feed intake and feed conversion ratio. Here, we built quadratic RSM models linking ME and CP to BWG and F/G at each growth stage, and we used multiple-response optimization to find the best nutrient combinations. RSM achieved reasonably high R^2^ values for F/G, suggesting it is reliable for this purpose. A limitation of the RSM analysis is the high multicollinearity arising from uncentered polynomial terms. While this does not bias the predicted response surface or the location of the optimum within the tested range, it means individual coefficient estimates are unstable, and their *p*-values should be interpreted as descriptive rather than as strict hypothesis tests. The overall model significance and lack-of-fit test remain valid. We then introduced ANN to see whether it could improve on RSM’s predictions.

A growing body of work shows that machine learning can outperform traditional regression when multiple environmental and biological variables are involved [26]. It can handle complex datasets—phenotypic traits, behavioral patterns—and supports data-driven decisions that improve poultry health and productivity [27,28,29]. Machine learning has also been shown to help fine-tune feeding strategies. By analyzing growth rate, feed intake, and environmental data, it can improve FCR and cut waste [30]. Mehri [31] applied ANN to adjust feed formulas for quail chicks, achieving the highest body weight gain at 2995 kcal/kg ME (≈12.53 MJ/kg; conversion: 1 kcal = 4.184 kJ) and the lowest FCR at 3000 kcal/kg ME (≈12.55 MJ/kg). Growth performance and feed efficiency both improved significantly. Faridi et al. [32] showed that machine learning can enhance feed formula research and support feed mill optimization. They used a group method of a data handling neural network to predict carcass energy content and composition of broilers from dietary inputs. We applied this approach to model the ME–CP–performance relationships in Hy-Line Brown male chicks from 1 to 70 days. Each model showed strengths in specific stage–response combinations under the primary data partition: ANN achieved higher R^2^ for BWG at 22–56 d and for F/G at 1–21 d, while RSM was competitive or better on the other measures. The ME optima predicted by RSM fell at the upper boundary of the tested range across all three growth stages (12.13 MJ/kg for the first two stages and 11.92 MJ/kg for the third), indicating that the optimizer selected the endpoints because the response surface lacked curvature information in the ME dimension. These boundary values should not be interpreted as definitive nutritional levels but merely as the best estimates within the experimental range. In contrast, ANN identified interior optima for all three stages (11.89, 11.92, and 11.91 MJ/kg, respectively), which may reflect local curvature generated by nonlinear basis functions, thereby locating peaks even when the polynomial surface had not yet saturated. However, given the poor leave-2-treatments-out performance for BWG, the ANN-optimized ME values should be interpreted as model-derived estimates from a single data partition rather than robust optima.

The discrepancy between in-sample and cross-validated performance for ANN likely reflects the small dataset size: with only 72 observations per stage across 12 treatment points, the network’s 21 trainable parameters can absorb noise rather than signal, whereas the polynomial’s inherent rigidity acts as a form of regularization that helps under sparse data. No fixed random seed was used for the primary ANN results (Table 5 and Table 6); however, the LOOCV and 200-iteration bootstrap analyses (Table 7) do not depend on any single seed, providing robust evidence for this study’s conclusions.

Future work should include more treatment groups and explore ensemble or hybrid modeling strategies. This would allow a more thorough evaluation of how different tools perform in precision feeding of Hy-Line Brown male chicks.

### 4.4. Limitations and Challenges

Several limitations affect the practical use of these models. First, no independent feeding trial has been conducted to validate the model-optimized dietary levels. A model trained on data from a single experimental facility may not transfer directly to diverse commercial settings [10,33]. The ANN is a “black box” with no physically interpretable parameters [34], and our dataset of 72 observations is small by ANN standards; the test set contained only two treatment combinations. Given that neural networks are known to overfit on small datasets [28,35], the optimized ME and CP levels should be viewed as experimental estimates, not universal standards [36,37]. Multi-farm validation is needed before these values can be applied commercially [10,33]. The modest R^2^ values (0.446–0.645 for BWG; 0.403–0.889 for F/G) also suggest that factors beyond ME and CP—individual bird variation [30], micro-environmental gradients and cage position within the house [26], and unmeasured dietary components [38]—account for a substantial share of the variance in growth.

The methodological divergence between the 1|group and 1|cage models (Section 4.2, Table A4) highlights a broader limitation: the choice of random-effects structure can qualitatively change diet–effect conclusions in longitudinal feeding trials. We recommend that future studies routinely report both specifications.

In this study, dietary energy and crude protein levels were adjusted by varying the proportions of corn, soybean meal, wheat bran, and corn gluten meal, with supplemental methionine and lysine added but no attempt made to balance the remaining amino acids. Nor were changes in other nutrient fractions, including ether extract, starch, and crude fiber, tracked, and neither amino acid supply nor nutrient digestibility was determined across treatments. Because these confounding variables move together with energy and protein when conventional ingredients are reformulated, it is hard to pull apart the independent contribution of metabolizable energy from the effects of the accompanying shifts in starch, fat, or fiber [39]. By the same token, the drops in body weight gain and feed conversion efficiency seen at lower CP cannot be pinned squarely on protein supply; they may just as well stem from shortfalls in specific amino acids such as arginine or valine that were not supplemented [40,41]. The model-estimated dietary levels reported here should, therefore, be read as practical dietary energy and protein levels under commercial feeding conditions, not as independent nutrient thresholds. Even so, the prediction approach itself still gives nutritionists a workable framework for optimizing ME and CP within the constraints of standard ingredient-based formulation.

Future work should include more treatment combinations and explore ensemble or hybrid modeling strategies, as well as collecting data from multiple commercial farms to build models with broader applicability. As smart livestock technologies advance, integrating ANN models with IoT sensors and real-time monitoring systems is a promising direction for refinement [42]. IoT systems can continuously stream environmental data (temperature, humidity, air quality), behavioral metrics (activity, feeding patterns), and feed intake records. Feeding these real-time data streams into AI models could reduce environmental noise, sharpen predictions, and enable dynamic, intelligent nutritional management during the rearing period [10].

## 5. Conclusions

Through systematic feeding trials, we developed RSM and ANN models that predict BWG and F/G from dietary ME and CP levels across three growth stages of Hy-Line Brown male chicks. The 22–56-day stage yielded the best model performance overall. For F/G, both models achieved strong and comparable fit (in-sample R^2^ ≈ 0.86–0.88) with LOOCV retention rates of 97%, and both remained predictive under leave-2-treatments-out validation (RSM R^2^ = 0.72, ANN R^2^ = 0.45). For BWG, model performance was weaker, and ANN’s apparent in-sample advantage over RSM (R^2^ = 0.585 vs. 0.388 at 22–56 d) did not hold under leave-2-treatments-out validation (ANN R^2^ = −12.7 vs. RSM R^2^ = −0.24), indicating limited generalizability given the small number of treatment combinations. The two approaches proved complementary: ANN identified interior ME–CP optima within the tested range for all three stages, though its generalization to BWG was limited under leave-2-treatments-out cross-validation, so these optima should be regarded as model-derived estimates rather than confirmed nonlinear relationships, whereas RSM was more robust under sparse-data conditions and provided interpretable coefficient estimates. Multi-objective optimization combining both models predicted stage-specific nutrient combinations of ME = 11.89–12.13 MJ/kg with CP = 19.50–20.0% (1–21 d), ME = 11.92–12.13 MJ/kg with CP = 18.0–18.27% (22–56 d), and ME = 11.91–11.92.

MJ/kg with CP = 16.83–16.84% (57–70 d). These findings provide a methodological framework—pairing the interpretability and stability of RSM with the nonlinear flexibility of ANN—for establishing stage-specific nutritional standards for Hy-Line Brown male chicks.

## Figures and Tables

**Table 1:** Experimental design of energy and protein nutrition levels for Hy-Line Brown male chicks.

Feeding Period	1 to 21 Days of Age		22 to 56 Days of Age		57 to 70 Days of Age	
Groups	ME/(MJ/kg)	CP %	ME/(MJ/kg)	CP %	ME/(MJ/kg)	CP %
1	12.13	20	12.13	19	11.92	18
2	11.92	20	11.92	19	11.72	18
3	11.72	20	11.72	19	11.51	18
4	11.51	20	11.51	19	11.30	18
5	12.13	19	12.13	18	11.92	17
6	11.92	19	11.92	18	11.72	17
7	11.72	19	11.72	18	11.51	17
8	11.51	19	11.51	18	11.30	17
9	12.13	18	12.13	17	11.92	16
10	11.92	18	11.92	17	11.72	16
11	11.72	18	11.72	17	11.51	16
12	11.51	18	11.51	17	11.30	16

**Table 2:** Composition and nutrient levels of experimental diets (air-dry basis) of 1 to 21 days%.

Items	1	2	3	4	5	6	7	8	9	10	11	12
Ingredients												
Corn	64.49	61.88	59.27	56.66	66.73	64.72	62.11	59.50	68.93	67.16	64.38	61.60
Soybean meal	27.34	27.11	26.88	26.65	26.39	27.85	27.62	27.39	25.27	27.45	26.74	26.03
Wheat bran	0.12	3.26	6.40	9.55	0.00	1.88	5.03	8.17	0.00	1.35	4.86	8.36
Corn protein meal	4.12	3.83	3.55	3.26	2.86	1.58	1.30	1.01	1.70	0.00	0.00	0.00
Limestone	1.70	1.79	1.86	1.95	1.68	1.73	1.80	1.90	1.65	1.70	1.79	1.87
CaHPO4	0.83	0.72	0.62	0.51	0.87	0.79	0.68	0.57	0.91	0.83	0.71	0.60
DL-Met	0.07	0.08	0.09	0.09	0.11	0.12	0.13	0.13	0.15	0.16	0.16	0.17
L-Lyshydrochloride	0.00	0.00	0.00	0.00	0.03	0.00	0.00	0.00	0.06	0.02	0.03	0.04
Salt	0.33	0.33	0.33	0.33	0.33	0.33	0.33	0.33	0.33	0.33	0.33	0.33
Premix ^1^	1.00	1.00	1.00	1.00	1.00	1.00	1.00	1.00	1.00	1.00	1.00	1.00
Total	100.00	100.00	100.00	100.00	100.00	100.00	100.00	100.00	100.00	100.00	100.00	100.00
Nutrient levels ^2^												
ME/(MJ/kg)	12.13	11.92	11.72	11.51	12.13	11.92	11.72	11.51	12.13	11.92	11.72	11.51
CP	20.00	20.01	19.98	20.02	19.03	19.00	18.98	19.01	18.05	17.93	18.04	18.00
Ca	1.00	0.98	1.02	1.00	1.02	0.98	1.00	1.01	1.00	0.99	1.01	1.00
TP	0.50	0.49	0.50	0.50	0.49	0.50	0.51	0.48	0.50	0.52	0.50	0.48
Met + Cys	0.74	0.72	0.74	0.72	0.73	0.73	0.72	0.71	0.73	0.71	0.71	0.72
Lys	1.00	1.02	1.00	0.98	1.00	1.02	0.97	1.00	1.00	0.98	1.01	1.00
Thr	0.76	0.75	0.75	0.76	0.76	0.76	0.77	0.75	0.76	0.75	0.75	0.74

^1^ The premix provided the following per kg of diets: VA 8000 IU, VB_1_ 4 mg, VB_2_ 3.6 mg, VB_5_ 40 mg, VB_6_ 4 mg, VB_12_ 0.02 mg, VD_3_ 3000 IU, VE 20 IU, VK_3_ 2 mg, biotin 0.15 mg, folic acid 1.0 mg, D-pantothenic acid 11 mg, nicotinic acid 10 mg, antioxidant 100 mg, Cu (as copper sulfate) 10 mg, Fe (as ferrous sulfate) 80 mg, Mn (as manganese sulfate) 80 mg, Zn (as zinc sulfate) 75 mg, I (as potassium iodide) 0.40 mg, Se (as sodium selenite) 0.30 mg, phytase 5000 FTU. ^2^ ME was a calculated value obtained according to the Chinese Feed Composition and Nutritional Value Table (33rd edition, 2022), while the others were measured values. The other nutrient contents were determined according to GB/T 6432—2018, GB/T 6436—2018, GB/T 6437—2018, and GB/T 18246—2019, respectively [13,14,15,16]. The RSM and ANN models used formulated/target values (Table 1) for both ME and CP as inputs, not the measured values shown here.

**Table 3:** Effects of dietary metabolizable energy and crude protein on growth performance of Hy-Line Brown male chicks.

Items	BWG (g) 1–21	F/G (g/g) 1–21		BWG (g) 22–56	F/G (g/g) 22–56		BWG (g) 57–70	F/G (g/g) 57–70
1	172.87	2.06		706.21 ^b^	2.79 ^f^		326.23	3.39 ^e^
2	174.00	2.06		696.08 ^b^	2.82 ^e^		320.40	3.49 ^d^
3	172.89	2.09		699.05 ^b^	2.88 ^d^		320.95	3.55 ^c^
4	170.10	2.09		687.20 ^c^	2.91 ^cd^		322.43	3.55 ^c^
5	172.49	2.06		704.71 ^b^	2.76 ^f^		318.75	3.33 ^e^
6	174.50	2.06		724.14 ^a^	2.80 ^d^		324.90	3.43 ^d^
7	173.66	2.06		685.52 ^c^	2.87 ^d^		326.00	3.53 ^c^
8	171.95	2.10		693.47 ^bc^	2.94 ^bc^		314.55	3.69 ^b^
9	168.05	2.09		686.54 ^c^	2.94 ^bc^		314.87	3.46 ^d^
10	168.60	2.13		697.89 ^b^	2.96 ^bc^		315.78	3.56 ^c^
11	168.39	2.11		676.06 ^d^	3.00 ^b^		299.30	3.72 ^b^
12	169.15	2.10		679.48 ^d^	3.17 ^a^		299.01	3.85 ^a^
SEM	2.43	0.03		9.88	0.04		10.72	0.08
Main effect								
ME/(MJ/kg)								
11.51	170.40	2.10	11.51	686.72	3.01	11.30	312.00	3.70
11.72	171.64	2.09	11.72	686.88	2.92	11.51	315.42	3.61
11.92	172.36	2.08	11.92	706.04	2.86	11.72	320.36	3.49
12.13	171.14	2.07	12.13	699.15	2.83	11.92	319.95	3.39
CP (%)								
18	168.55 ^b^	2.11 ^a^	17	684.99	3.02	16	307.24 ^b^	3.64
19	173.15 ^a^	2.07 ^b^	18	701.96	2.84	17	321.05 ^a^	3.49
20	172.46 ^a^	2.07 ^b^	19	697.13	2.85	18	322.50 ^a^	3.50
*p*-Value								
ME	0.1125	0.1225		<0.0001	<0.0001		0.0698	<0.0001
CP	<0.0001	0.0003		<0.0001	<0.0001		<0.0001	<0.0001
ME × CP	0.357	0.149		0.0003	0.0018		0.0836	0.0124

When the interaction effect is significant, values in the same column sharing a common letter indicate no significant difference (*p* > 0.05), whereas different lowercase letters indicate a significant difference (*p* < 0.05). When the interaction effect is not significant, no multiple comparisons among treatment combinations were performed.

**Table 4:** Quadratic polynomial response surface model analysis between performance indices and dietary energy and protein levels of Hy-Line Brown male chicks.

Days of Age	Items	Quadratic Polynomial Response Surface Model	*p*-Value	*p*_LOF
1 to 21 days of age	BWG	BWG_1–21_ = −3260 + 422.6ME + 95.6CP − 18.12ME^2^ − 2.58CP^2^ + 0.38ME × CP	<0.001	0.942
	F/G	F/G_1–21_ = 0.15 + 1.37ME − 0.60CP − 0.048ME^2^ + 0.020CP^2^ − 0.015ME × CP	<0.001	0.388
22 to 56 days of age	BWG	BWG_22–56_ = −7418 + 819.9ME + 340.3CP − 35.74ME^2^ − 10.23CP^2^ + 2.88ME × CP	<0.001	0.614
	F/G	F/G_22–56_ = 107.5 − 10.86ME − 4.23CP + 0.39ME^2^ + 0.091CP^2^ + 0.074ME × CP	<0.001	0.234
57 to 70 days of age	BWG	BWG_57–70_ = −5534 + 330.5ME + 443.3CP − 3.97ME^2^ − 8.39CP^2^ − 12.95ME × CP	<0.001	0.633
	F/G	F/G_57–70_ = 65.81 − 3.5ME − 4.51CP + 0.02ME^2^ + 0.082CP^2^ + 0.143ME × CP	<0.001	0.443

RSM regression coefficients were estimated in Minitab (22.4.0) using all 72 cage-level observations (6 replicates × 12 treatments). The R^2^ and model fit statistics reported by Minitab (22.4.0) are based on these 72 observations. Some terms are not statistically significant due to high multicollinearity (VIF = 3000–17,000) in uncentered polynomial models. Response optima in Figure 2 were computed using full-precision coefficients via Minitab’s response optimizer, which maximizes composite desirability across both BWG and F/G.

**Table 5:** Pearson correlation coefficients (R) between predicted and observed growth performance at the cage level from ANN models.

Days of Age	Items	Training R	Validation R	Test R	All R
1 to 21 days of age	BWG	0.817	0.702	0.734	0.784
	F/G	0.673	0.555	0.624	0.651
22 to 56 days of age	BWG	0.885	0.756	0.788	0.804
	F/G	0.964	0.913	0.936	0.941
57 to 70 days of age	BWG	0.723	0.656	0.678	0.687
	F/G	0.963	0.907	0.914	0.925

ANN trained on 72 cage-level observations split by treatment: 8 groups (*n* = 48) training, 2 (*n* = 12) validation, 2 (*n* = 12) test. Values are Pearson correlation coefficients (R) between predicted and observed values. 2–5–1 architecture (21 trainable parameters), log-sigmoid transfer. No fixed random seed was used for the primary single-run results. All R is the weighted average of training, validation, and test set correlations, weighted by sample size.

**Table 6:** Comparison of parameters between the ANN model and the RSM model.

Days of Age	Model	Item	R^2^	MSE	MAE
1 to 21 days of age	RSM	BWG	0.624	0.258	0.448
		F/G	0.403	1.36 × 10^−4^	0.00985
	ANN	BWG	0.615	0.897	0.703
		F/G	0.42	1.34 × 10^−4^	0.0097
22 to 56 days of age	RSM	BWG	0.480	30.25	4.286
		F/G	0.862	3.3 × 10^−4^	0.015
	ANN	BWG	0.645	12.107	2.24
		F/G	0.885	2.75 × 10^−4^	0.0137
57 to 70 days of age	RSM	BWG	0.446	13.527	3.085
		F/G	0.848	5.05 × 10^−4^	0.0193
	ANN	BWG	0.472	4.748	0.959
		F/G	0.856	5.12 × 10^−4^	0.0194

RSM and ANN R2 in this table are both computed at the treatment-mean level (*n* = 12 design points) after fitting on 72 cage-level observations and are, therefore, directly comparable. The treatment-mean R2 is typically higher than the cage-level R2 (*n* = 72, reported in Table 7) because between-replicate variation is averaged out. ANN predictions are from the same cage-level model described in Table 5 (not refit on treatment means). Note that the R^2^ values in Table 6 are computed as the squared correlation between model predicted and observed values at the treatment-mean level (*n* = 12), consistent with standard RSM practice.

**Table 7:** Cross-validation summary (LOOCV and leave-2-treatments-out).

Stage	Response	Model	In-Sample R^2^	LOOCV R^2^	Retention (%)	Leave-2-Trt R^2^
1 to 21 days of age	RSM	BWG	0.486	0.380	78	0.376
		F/G	0.286	0.151	53	−0.104
	ANN	BWG	0.509	0.314	62	−1.402
		F/G	0.273	0.166	61	−0.501
22 to 56 days of age	RSM	BWG	0.388	0.282	73	−0.235
		F/G	0.862	0.838	97	0.721
	ANN	BWG	0.585	0.413	71	−12.682
		F/G	0.882	0.851	97	0.447
57 to 70 days of age	RSM	BWG	0.371	0.252	68	−0.237
		F/G	0.764	0.717	94	0.600
	ANN	BWG	0.449	0.212	47	−7.172
		F/G	0.781	0.700	90	0.649

In-sample R^2^ for both RSM and ANN is computed as the squared Pearson correlation between predicted and observed values across all 72 cage-level observations. For RSM, this cage-level R^2^ differs from the treatment-mean R^2^ reported in Table 6 (based on *n* = 12 design points) because cage-level R^2^ incorporates within-treatment replicate variation. LOOCV R^2^ is computed by leave-one-out cross-validation on the same 72 observations. Leave-2-trt R^2^ is computed by holding out all replicates of two treatment combinations (12 observations) and predicting them with a model trained on the remaining 60. Retention (%) = LOOCV R^2^/In-sample R^2^ × 100. The cage-level in-sample R^2^ (*n* = 72) is reported separately for cross-validation purposes.

## Data Availability

The datasets and code supporting the findings of this study are openly available in the Zenodo repository at https://doi.org/10.5281/zenodo.22840312. Further inquiries can be directed to the corresponding author.

## Supplementary Materials

The following supporting information can be downloaded at: https://doi.org/10.5281/zenodo.22840312, Table S1. Cage-level growth performance data for Hy-Line Brown male chicks across three growth stages (1–21 d, 22–56 d, 57–70 d). Each row represents one cage (*n* = 72 total; 6 replicates × 12 dietary treatments). Columns include treatment ID, ME level (MJ/kg), CP level (%), replicate number, body weight gain (BWG, g), and feed-to-gain ratio (F/G). The same Excel workbook (data_three_stages.xlsx) also contains the ANN training/validation/test split assignments, including the random seed-based allocation of the 12 dietary treatments to the training, validation, and test sets for ANN model development. File S1. code_S1_ann_nsga2.m. MATLAB scripts containing: (i) the data preprocessing (data cleaning, outlier detection, treatment-mean aggregation) and ANN model training code, implementing the feedforward neural network (MLP) architecture with hyperparameter tuning, early stopping, and model evaluation; and (ii) the Non-dominated Sorting Genetic Algorithm II (NSGA-II) code for constrained multi-objective optimization of ME and CP levels (simultaneously maximizing BWG and minimizing F/G), including Pareto front generation and optimal solution selection criteria. File S2. code_S2_mixed_model.R. R scripts for mixed model analysis of the cage-level growth performance data. File S3. code_S3_bootstrap_pareto.py. Python scripts for bootstrap resampling (*n* = 1000 iterations) and Pareto analysis to estimate 95% confidence intervals for RSM and ANN model predictions, R^2^ values, and optimal nutrient recommendations, including both cage-level and treatment-mean bootstrap procedures.

## Abbreviations

The following abbreviations are used in this manuscript:

ME	Metabolizable Energy
CP	Crude Protein
BWG	Body Weight Gain
F/G	Feed-to-Gain ratio
ANN	Artificial Neural Network
RSM	Response Surface Methodology
MSE	Mean Squared Error
MAE	Mean Absolute Error
R^2^	Coefficient of Determination
LOOCV	Leave-One-Out Cross-Validation
NSGA-II	Non-dominated Sorting Genetic Algorithm II
HSD	Honestly Significant Difference
SEM	Standard Error of the Mean
ANOVA	Analysis of Variance
GLM	General Linear Model

## Appendix A

**Table A1:** Composition and nutrient levels of experimental diets (air-dry basis) of 22 to 56 days %.

Items	1	2	3	4	5	6	7	8	9	10	11	12
Ingredients												
Corn	67.24	64.94	62.33	59.72	69.44	67.71	64.93	62.15	71.63	69.20	66.42	63.64
Soybean meal	21.27	21.90	21.67	21.45	20.16	22.43	21.72	21.00	19.05	19.34	18.63	17.91
Cottonseed meal	5.00	5.00	5.00	5.00	5.00	5.00	5.00	5.00	5.00	5.00	5.00	5.00
Wheat bran	0.00	2.50	5.64	8.79	0.00	1.28	4.78	8.29	0.00	2.76	6.26	9.77
Corn protein meal	2.92	2.13	1.84	1.56	1.75	0.00	0.00	0.00	0.59	0.00	0.00	0.00
Limestone	1.68	1.74	1.83	1.90	1.65	1.70	1.79	1.88	1.63	1.71	1.80	1.89
CaHPO4	0.47	0.38	0.27	0.16	0.51	0.43	0.32	0.20	0.55	0.44	0.33	0.21
DL-Met	0.07	0.08	0.09	0.09	0.11	0.12	0.12	0.13	0.14	0.15	0.15	0.16
L-Lyshydrochloride	0.02	0.00	0.00	0.00	0.05	0.00	0.01	0.02	0.08	0.07	0.08	0.09
Salt	0.33	0.33	0.33	0.33	0.33	0.33	0.33	0.33	0.33	0.33	0.33	0.33
Premix ^1^	1.00	1.00	1.00	1.00	1.00	1.00	1.00	1.00	1.00	1.00	1.00	1.00
Total	100.00	100.00	100.00	100.00	100.00	100.00	100.00	100.00	100.00	100.00	100.00	100.00
Nutrient levels ^2^												
ME/(MJ/kg)	12.13	11.92	11.72	11.51	12.13	11.92	11.72	11.51	12.13	11.92	11.72	11.51
CP	19.00	19.08	18.86	19.09	18.00	17.96	18.06	18.07	17.00	17.17	16.93	17.05
Ca	0.88	0.90	0.91	0.88	0.91	0.89	0.90	0.90	0.89	0.90	0.92	0.89
TP	0.46	0.44	0.45	0.46	0.45	0.45	0.44	0.45	0.44	0.45	0.45	0.45
Met + Cys	0.68	0.71	0.70	0.70	0.69	0.71	0.69	0.73	0.70	0.69	0.69	0.70
Lys	0.95	0.93	0.94	0.91	0.95	0.95	0.92	0.94	0.94	0.95	0.92	0.91
Thr	0.74	0.72	0.73	0.75	0.69	0.71	0.69	0.69	0.65	0.64	0.65	0.63

^1^ Same definition as in Table 2. ^2^ Same definition as in Table 2.

**Table A2:** Composition and nutrient levels of experimental diets (air-dry basis) of 57 to 70 days %.

Items	1	2	3	4	5	6	7	8	9	10	11	12
Ingredients												
Corn	66.55	63.95	61.36	58.76	68.89	66.11	63.35	60.60	70.39	67.61	64.84	62.09
Soybean meal	19.70	19.48	19.26	19.04	19.01	18.30	17.61	16.92	15.92	15.21	14.52	13.83
Cottonseed meal	5.00	5.00	5.00	5.00	5.00	5.00	5.00	5.00	5.00	5.00	5.00	5.00
Wheat bran	4.16	7.28	10.36	13.44	3.84	7.35	10.78	14.21	5.32	8.83	12.26	15.67
Corn protein meal	1.41	1.13	0.85	0.57	0.00	0.00	0.00	0.00	0.00	0.00	0.00	0.00
Limestone	1.69	1.74	1.75	1.76	1.65	1.75	1.76	1.76	1.66	1.75	1.78	1.80
CaHPO4	0.08	0.00	0.00	0.00	0.13	0.01	0.00	0.00	0.14	0.02	0.00	0.00
DL-Met	0.08	0.09	0.09	0.10	0.12	0.12	0.13	0.13	0.15	0.15	0.16	0.16
L-Lyshydrochloride	0.00	0.00	0.00	0.00	0.03	0.03	0.04	0.05	0.09	0.10	0.11	0.12
Salt	0.33	0.33	0.33	0.33	0.33	0.33	0.33	0.33	0.33	0.33	0.33	0.33
Premix ^1^	1.00	1.00	1.00	1.00	1.00	1.00	1.00	1.00	1.00	1.00	1.00	1.00
Total	100.00	100.00	100.00	100.00	100.00	100.00	100.00	100.00	100.00	100.00	100.00	100.00
Nutrient levels ^2^												
ME/(MJ/kg)	11.92	11.72	11.51	11.30	11.92	11.72	11.51	11.30	11.92	11.72	11.51	11.30
CP	18.00	18.08	17.95	18.03	17.00	16.98	17.08	16.95	16.00	15.98	16.04	15.97
Ca	0.80	0.79	0.82	0.81	0.79	0.82	0.79	0.80	0.81	0.78	0.80	0.82
TP	0.40	0.40	0.42	0.44	0.40	0.40	0.42	0.44	0.40	0.40	0.42	0.44
Met + Cys	0.67	0.65	0.69	0.64	0.68	0.67	0.65	0.68	0.67	0.69	0.66	0.67
Lys	0.90	0.87	0.93	0.88	0.91	0.90	0.89	0.91	0.89	0.92	0.88	0.90
Thr	0.69	0.68	0.68	0.67	0.65	0.64	0.64	0.63	0.60	0.60	0.59	0.59

^1^ Same definition as in Table 2. ^2^ Same definition as in Table 2.

**Table A3:** Mean initial body weight (g) of chicks by treatment group.

Items	1	2	3	4	5	6	7	8	9	10	11	12
Mean Initial BW (g)	38.05	37.96	38.12	37.89	38.02	37.98	38.15	37.85	37.92	38.08	37.82	37.99
SEM	0.21	0.19	0.23	0.20	0.22	0.18	0.24	0.21	0.19	0.22	0.20	0.21

**Table A4:** Mixed-model analysis of stage-specific diet effects and stage interactions in the longitudinal feeding design.

Panel A: Stage-specific *p*-values for ME and CP under four model specifications										
Stage	Response	Without prior control		With prior-stage covariate (OLS)			Mixed model (1|group)		Mixed model (1|cage)	
		ME *p*	CP *p*	ME *p*	CP *p*	Prior cov. *p*	ME *p*	CP *p*	ME p	CP *p*
22 to 56 days of age	BWG	0.874	0.783	0.845	0.944	0.087	0.936	0.894	0.03	0.05
	F/G	0.030	0.049	0.011	0.018	0.024	0.283	0.326	<0.001	<0.001
57 to 70 days of age	BWG	0.053	0.056	0.031	0.029	0.0003	0.094	0.088	0.009	<0.001
	F/G	0.0003	0.001	0.002	0.005	0.019	0.007	0.015	<0.001	<0.001
Panel B: Interaction term tests (Model A, long-format mixed model, all 3 stages)										
Response	ME × stage				CP × stage					
	F	df		*p*	F	df	*p*			
BWG	7.46	2, 206		0.001	4.84	2, 206	0.009			
F/G	9.68	2, 133		<0.001	8.64	2, 133	<0.001			
Panel C: Interaction tests with prior covariate (Model B, stages 2–3)										
Response	ME × stage				CP × stage					
	F	df		*p*	F	df	*p*			
BWG	1.35	1, 136		0.247	0.71	1, 136	0.402			
F/G	2.24	1, 136		0.137	4.20	1, 136	0.042			
Panel D: Three-way interaction test (ME × CP × stage, Model A)										
Response	ME × CP × stage									
	F	df		*p*						
BWG	2.57	2, 133		0.08						
F/G	8.35	2, 133		<0.001						

Panel A: Stage-specific *p*-values for ME and CP effects under four model specifications. Without prior control: OLS ANOVA. With prior-stage covariate (OLS): OLS ANCOVA including preceding stage performance. Mixed model (1|group): wide-format mixed model with treatment group (*n* = 12) as random intercept. Mixed model (1|cage): long-format mixed model with cage (*n* = 72) as repeated-measures subject, compound symmetry covariance, all 216 observations (ICC = 0.21 for BWG, 0.19 for F/G). Kenward-Roger degrees of freedom. Panel B: Wald F-tests for interaction terms from Model A (all 3 stages). Panel C: Interaction tests from Model B (stages 2–3 with prior covariate). For BWG, Model A uses the reduced specification (without ME × CP × stage, which was non-significant at *p* = 0.080). For F/G, Model A retains the ME × CP × stage interaction (significant at *p* < 0.001; F(2, 133) = 8.35; see Panel D); accordingly, the ME × stage and CP × stage F-values for F/G are computed from the full model. All Panel B interactions are significant (*p* < 0.01), confirming diet effects differ across stages. Panel D: The F/G mixed model (1|cage) retains the ME × CP × stage three-way interaction (*p* < 0.001), so stage-specific ME and CP effects for F/G are simple main effects evaluated at stage-specific ME/CP means. The BWG mixed model does not include the three-way interaction (*p* = 0.080).

**Table A5:** Complete Specification Table of ANN.

Parameter	Setting	Notes
Architecture	2–5–1 feedforward	Same for all 6 networks (3 stages × 2 outcomes)
Input neurons	2 (ME, CP)	Calculated formulation values
Hidden neurons	5	Single hidden layer
Output neuron	1 (BWG or F/G)	Separate network per outcome
Hidden transfer function	Log-sigmoid (logsig)	MATLAB(R2022b) default for hidden layer
Output transfer function	Log-sigmoid (logsig)	MATLAB(R2022b) default for output layer
Trainable parameters	21 (15 weights + 6 biases)	Per network
Training algorithm	Levenberg–Marquardt (trainlm)	MATLAB(R2022b) default; fast convergence on small datasets
Data split	8–2–2 by treatment	48 training/12 validation/12 test; all reps of a treatment in same subset
Input scaling	Normalization (mapminmax)	Inputs scaled to [−1, 1] before training; outputs inverse-transformed
Initialization	Nguyen-Widrow (initnw)	MATLAB(R2022b) default for log-sigmoid layers
Epoch limit	1000 (default)	MATLAB(R2022b) default maximum
Early stopping	Validation set performance	Training stops when validation MSE stops improving for 6 consecutive epochs (default)
Performance metric	MSE (mean squared error)	Training objective
Minimum gradient	1 × 10^−7^ (default)	Training stops if gradient falls below threshold
Mu (initial)	0.001 (default)	Levenberg–Marquardt damping parameter
Maximum mu	1 × 10^10^ (default)	Training stops if μ exceeds this value

**Table A6:** Coefficient estimates, standard errors, hypothesis tests, 95% confidence intervals, and variance inflation factors for the RSM.

Stage	Response	Term	Estimate	SE	t	*p*	95% CI Lower	95% CI Upper	VIF
1–21 d	BWG	Constant	−3259.52	824.99	−3.95	<0.001	−4909.75	−1609.2943	-
		ME	422.55	126.99	3.33	0.0015	168.5373	676.5648	16,587.1
		CP	95.58	23.81	4.02	<0.001	47.9598	143.2009	7350
		ME^2^	−18.12	5.23	−3.47	<0.001	−28.5793	−7.6667	15,719.2
		CP^2^	−2.58	0.47	−5.46	<0.001	−3.5215	−1.6327	4177
		ME × CP	0.38	1.25	0.3	0.7626	−2.1206	2.8791	3264.9
	F/G	Constant	0.15	12	0.0125	0.9904	−23.8358	24.1261	-
		ME	1.37	1.87	0.73	0.4658	−2.3666	5.1117	16,689.3
		CP	−0.6	0.35	−1.73	0.0885	−1.2936	0.0931	7494.7
		ME^2^	−0.048	0.08	−0.62	0.535	−0.2032	0.1065	16,004
		CP^2^	0.02	0.007	2.76	0.0077	0.0055	0.0343	4689.2
		ME × CP	−0.015	0.018	−0.84	0.4042	−0.0499	0.0204	3166.7
22–56 d	BWG	Constant	−7417.91	5046.61	−1.47	0.1463	−17,493.7976	2657.9723	-
		ME	819.87	802.83	1.02	0.3109	−783.0379	2422.782	16,483.8
		CP	340.31	142.46	2.39	0.0198	55.8734	624.7377	6522.6
		ME^2^	−35.74	33.46	−1.07	0.2892	−102.5389	31.0529	15,997.8
		CP^2^	−10.23	3.03	−3.36	0.0013	−16.3305	−4.1305	3889
		ME × CP	2.88	7.66	0.38	0.7079	−12.4088	18.1725	3120.6
	F/G	Constant	107.52	18.35	5.85	<0.001	70.8757	144.1609	-
		ME	−10.858	2.91	−3.71	<0.001	−16.6871	−5.0286	16,483.8
		CP	−4.233	0.51	−8.17	<0.001	−5.2673	−3.1985	6522.6
		ME^2^	0.391	0.12	3.21	0.002	0.1481	0.6339	15,997.8
		CP^2^	0.091	0.011	8.18	<0.001	0.0688	0.1131	3889
		ME × CP	0.074	0.03	2.65	0.0099	0.0184	0.1296	3120.6
57–70 d	BWG	Constant	−5533.84	4109	−1.35	0.1828	−13,742.5206	2674.8373	-
		ME	330.55	676.04	0.49	0.6265	−1019.9955	1681.0938	16,071.1
		CP	443.26	116.14	3.82	<0.001	211.2496	675.2626	6215.8
		ME^2^	−3.97	28.75	−0.14	0.8906	−61.4048	53.4618	15,660.1
		CP^2^	−8.39	2.63	−3.19	0.0022	−13.6527	−3.1269	3698.5
		ME × CP	−12.95	6.36	−2.04	0.046	−25.6636	−0.2348	2930
	F/G	Constant	65.81	24.91	2.64	0.0104	16.0213	115.5918	-
		ME	−3.503	4.1	−0.85	0.3966	−11.7057	4.6994	15,776.2
		CP	−4.512	0.7	−6.41	<0.001	−5.9184	−3.1047	6109.6
		ME^2^	0.0247	0.1751	0.141	0.8884	−0.3253	0.3747	15,482.1
		CP^2^	0.082	0.016	5.147	<0.001	0.0504	0.1143	3648.6
		ME × CP	0.143	0.04	3.63	<0.001	0.0629	0.2201	3040.8

Models were fitted on cage-level observations using full second-order polynomials (Constant + ME + CP + ME^2^ + CP^2^ + ME × CP). All terms were retained regardless of statistical significance. VIF = variance inflation factor. The high VIF values (2930–16,700) reflect structural multicollinearity inherent to uncentered polynomial regression and do not affect the fitted response surface or predicted optima within the design space. 95% CI = estimate ± t(0.025, df) × SE.

**Table A7:** Mortality and final bird counts for each treatment across three feeding stages.

Treatment	1–21 d			22–56 d			57–70 d			Total Dead	Mortality (%)
	Start	Dead	End	Start	Dead	End	Start	Dead	End		
**1**	180	2	178	178	1	177	177	0	177	3	1.67
**2**	180	1	179	179	1	178	178	1	177	3	1.67
**3**	180	2	178	178	1	177	177	0	177	3	1.67
**4**	180	1	179	179	0	179	179	0	179	1	0.56
**5**	180	0	180	180	1	179	179	1	178	2	1.11
**6**	180	2	178	178	1	177	177	0	177	3	1.67
**7**	180	1	179	179	1	178	178	0	178	2	1.11
**8**	180	2	178	178	0	178	178	1	177	3	1.67
**9**	180	1	179	179	1	178	178	0	178	2	1.11
**10**	180	0	180	180	1	179	179	0	179	1	0.56
**11**	180	1	179	179	1	178	178	1	177	3	1.67
**12**	180	1	179	179	1	178	178	1	177	3	1.67
**Total**	2160	14	2146	2146	10	2136	2136	5	2131	29	1.34

Note: Each treatment had 6 replicate cages of 30 birds (180 birds per treatment; 2160 birds total). Birds that died were not replaced. No entire cage was lost; all 72 cages contributed data to all three stages. Overall mortality across the full trial (1–70 d) was 1.34% (29/2160).

**Table A8:** Pareto compromise solution bootstrap stability.

Stage	Pareto Non-Dominated Set	Full-Data Compromise	Compromise ME 95% CI	Compromise CP 95% CI	Compromise Selection Frequency (%)
1–21 d	G2, G5, G6	G2 (11.92, 20%)	[11.72, 12.13]	[19.00, 20.00]	24.5
22–56 d	G1, G5, G6	G6 (11.92, 18%)	[11.92, 11.92]	[18.00, 18.00]	98.0
57–70 d	G1, G5	G1 (11.92, 18%)	[11.50, 11.92]	[17.00, 18.00]	57.5
